# Mechanical and dose delivery accuracy evaluation in radiosurgery using polymer gels

**DOI:** 10.1120/jacmp.v7i4.2273

**Published:** 2006-11-28

**Authors:** Panagiotis Sandilos, Elias Tatsis, Lampros Vlachos, Constantinos Dardoufas, Pantelis Karaiskos, Evangelos Georgiou, Panagiotis Baras, Panagiotis Kipouros, Michael Torrens, Angelos Angelopoulos

**Affiliations:** ^1^ Department of Radiology, Medical School University of Athens, Areteion Hospital 76 Vas. Sofias Ave., 115 28 Athens; ^2^ Medical Physics Department, Medical School University of Athens 75 Mikras Asias, 115 27 Athens; ^3^ Philips Hellas Medical Systems 44 Kifissias Ave., Maroussi 151 25 Athens; ^4^ Greek Atomic Energy Commission Ag. Paraskevi, 153 10, PO Box 60092 Athens; ^5^ Radiosurgery Department Hygeia Hospital Kiffisias Avenue and 4 Erythrou Stavrou, Marousi, 151 23 Athens; ^6^ Nuclear and Particle Physics Section, Physics Department University of Athens Panepistimioupolis, Ilisia, 157 71 Athens Greece

**Keywords:** stereotactic radiosurgery, gamma knife, gel dosimetry

## Abstract

The polymer gel–magnetic resonance imaging (MRI) dosimetry technique was used to evaluate the mechanical and dose delivery accuracy in Leksell gamma‐knife stereotactic radiosurgery for the treatment of multiple targets. Two different polymer gel dosimeter formulations reported in the literature were prepared in‐house. A plan for the treatment of four brain metastases (targets) was generated. It involved the delivery of four 8‐mm collimator shots using different prescription isodose lines and different prescription doses for each target, keeping the maximum dose constant for all targets. A sample of each gel formulation was irradiated using a custom‐made phantom with an experimental procedure capable of testing the increased nominal mechanical accuracy of stereotactic radiosurgery. The irradiated dosimeters were evaluated using a clinical 1.5 T MR imager. Result manipulation in 3D allowed for the determination of the mechanical accuracy in the delivery of each shot through the comparison of measured versus planned shot center coordinates. Dose delivery accuracy was also evaluated by comparison of maximum dose values measured at the center of each shot as well as dose distribution measurements, with corresponding treatment‐planning calculations. Polymer gel dosimetry was found capable of verifying the complete chain of radiosurgery treatment in gamma‐knife applications involving the irradiation of multiple targets.

PACS numbers: 87.53.Dq, 87.53.Ly, 87.53.Xd

## I. INTRODUCTION

Stereotactic gamma‐knife radiosurgery (Elekta AB, Stockholm, Sweden) is widely used as an alternative to surgical resection in the treatment of brain metastases due to its advantages in terms of cost, hospitalization, morbidity, mortality, and wider applicability. This technique uses 201 C60o beams intersecting at the so‐called unit center point (UCP) and four collimator helmets that form nominal beam sizes of diameter 18 mm, 14 mm, 8 mm, or 4 mm at the UCP. For patient treatment, a stereotactic frame is attached to the patient's head, which establishes a 3D coordinate system for the accurate determination of target locations through imaging. The high mechanical and target localization accuracy, along with the presence of very steep dose gradients in all three dimensions, facilitates precise delivery of high dose to the treated target volumes while maintaining the dose to the adjacent healthy tissue within the accepted tolerance levels.^(^ ^1^ ^,^ ^2^ ^)^

Brain metastases are an attractive target for gamma‐knife radiosurgery because they are clearly delineated with MRI, and they are, typically, spherical in shape, so that they can usually be treated using a single shot. The introduction of an automated positioning system in the later commercially available model (Leksell Gamma Knife model C) to reduce delivery time enhances the need for a mechanical accuracy verification procedure. Moreover, treatment planning using dedicated software (GammaPlan release 5.34) is performed in a cubic matrix of 31×31×31 points (maximum matrix size is $7.5\times 7.5\times 7.5\;{\text{cm}}^{3}$). For multiple target treatments, where targets are usually away from each other and cannot be included in the same matrix or different prescription doses have to be ascribed to different targets, dose is calculated in multiple matrixes. A special procedure is followed that takes into account the dose contribution in a matrix from shots belonging to other matrixes and allows for different prescription isodoses (those encompassing the target volume) and different prescription doses for the different treated targets.

In order to verify this procedure, as well as the overall accuracy of the technique when treating multiple targets, an experimental method based on the polymer gel–MRI method was used. This method simulates the entire patient treatment and presents the unique advantage of providing 3D dose distribution measurements with high resolution^(^ ^3^ ^–^ ^6^ ^)^ in a water‐equivalent material,^(7)^ thus allowing for the experimental verification of the 3D steep dose gradients met in gamma‐knife applications of single‐ or multishot applications to a single target.^(^ ^8^ ^–^ ^11^ ^)^ Two different polymer gel dosimeter formulations (PABIG and VIPAR) were used. A treatment of four brain metastases (targets) was simulated involving the delivery of four 8‐mm collimator shots with the same maximum dose for each target (20 Gy for PABIG and 30 Gy for VIPAR formulation), while a different prescription isodose line (45% to 80%) covered each target. Experimental results were compared to corresponding GammaPlan calculations in the form of shot center coordinates, dose delivered to the center of each shot, and relative dose distributions.

## II. METHODS AND MATERIALS

### A. Gel preparation

VIPAR (4% w/v 1‐vinyl‐2‐pyrrolidone, 4% w/v *N*,*N*‐ methylenebisacrylamide, 5% w/v gelatin) and PABIG gel (4% w/v polyethylene glycol diacrylate, 4% w/v *N,N*–methylenebisacrylamide, 5% w/v gelatin) were prepared in‐house along the lines of Baldock et al.,^(12)^ as described elsewhere.^(^ ^10^ ^,^ ^11^ ^)^ Major points in the manufacturing procedure include the purification of monomers before preparation and the implementation of a thorough deoxygenating procedure necessary to ensure the minimum possible oxygen presence in the gel. Both gel formulations exhibit comparable dose sensitivity and a wide linear dose‐response region extending up to about 40 Gy.^(^ ^6^ ^,^ ^13^ ^)^ The VIPAR gel also features an increased dynamic dose range of dose response (up to about 250 Gy) at the expense, however, of dose resolution in the low‐dose region.^(13)^

A Pyrex® cylindrical vial of 95 mm total height (with the screw top cap in place) and 47.5 mm inner diameter was filled with each gel formulation for the needs of gamma‐knife irradiation in this work. Another gel vial (100 mL volumetric flask) was filled with each gel formulation (calibration gel), with a flexible closed‐end catheter of 1.5 mm external diameter (Nucletron BV, the Netherlands) introduced through an appropriate hole drilled through the vial's cap to facilitate irradiation using a Nucletron microSelectron I192r high dose rate afterloader for the purpose of calibrating the dose response of the prepared PABIG and VIPAR gel batches.^(13)^

The gel‐filled vials were stored in the lab overnight at a room temperature of about 20 ±C, transferred to the radiotherapy department on the following day, and irradiated at approximately 23 ±C. One vial of each gel formulation was kept unirradiated to serve as a control for the zero dose reading in the MR readout session of irradiated gels. The density of both formulations under these temperature conditions and their percentage elemental composition by weight ensure water equivalence as a phantom/detector material for the C60o energies.^(7)^

### B. Treatment planning and irradiation

For dose distribution registration between measurements and treatment‐planning predictions, multimodality fiducial markers, visible on both CT and MR images, were used.^(^ ^10^ ^,^ ^11^ ^,^ ^14^ ^)^ Three markers, which are circular stickers of 1.5 cm diameter and 0.3 cm thickness with a circular hollow center of 0.5 cm diameter (MM 3005, IZI Medical Products Corp., Baltimore, MA), were adhered on predetermined positions of the gel vial using a custom‐built aluminum tool in order to establish a 3D coordinate system of reference. This coordinate system can be reconstructed in both the irradiation planning and readout phases of the experiment and thus facilitates the comparison of planned and measured dose distributions.

For the purpose of irradiation, each gel vial with fiducial markers in place was accommodated in a custom‐built, spherical (16.3 cm diameter) Plexiglas phantom. The Leksell stereotactic frame was attached to the phantom by fastening the four fixation screws on predetermined phantom positions so that the reference coordinate system defined by the fiducial markers lays aligned with the stereotactic coordinate system of the gamma‐knife unit. In this coordinate system, which is also used for reporting results in the following sections, the *x*‐axis is along the patient right–left direction, the *y*‐axis is along the patient posterior–anterior direction, and the *z*‐axis is along the patient cephalic–caudal direction.

An MR imaging session of a gel‐phantom–stereotactic frame assembly was performed on a 1.5 T whole‐body Philips ACS NT MR imager (Philips Medical Systems, Best, the Netherlands) with an RF quadrature receiver head coil, using MR sequences identical to the ones used for patient imaging (a spoiled T1‐weighted 3D‐Fast Field Echo sequence of TR: 25 ms/TE:1.8 ms/Flip Angle: 35±, and a T2‐weighted Turbo Spin Echo sequence of TR: 2700 ms/TE:160 ms/Flip Angle: 90±, 1.5 mm slice thickness, and $0.9\times 0.9\;{\text{mm}}^{2}$ in‐plane spatial resolution). The acquired images were imported to the GammaPlan treatment‐planning system (TPS) software, and a treatment plan resembling treatment of a multiple brain metastases treatment was generated. Four targets simulating four different metastases with volumes of 0.62 cm^3^ (target A1), 0.57 cm^3^ (target A2), 0.18 cm^3^ (target B), and 0.44 cm^3^ (target C) were drawn in different regions within the polymer gel volume. Each of these volumes was treated with a single 8‐mm collimator shot, using a prescription isodose line that covered the treated target (45% for targets A1 and A2, which belonged to the same TPS‐calculated dose matrix, 80% for target B, and 50% for target C, which belonged to two different TPS‐calculated dose matrixes; see also Fig. 2). The maximum dose was kept constant for all targets (20 Gy for the PABIG gel and 30 Gy for the VIPAR gel); thus, the prescription dose of each target varied from 9 Gy to 16 Gy for the PABIG formulation and from 13.5 Gy to 24 Gy for the VIPAR formulation. The phantom along with the attached stereotactic frame was mounted on the Leksell gamma knife® model 4200C, and the irradiation was performed using the automated positioning system.

Each calibration gel, stored under the same conditions with the gamma‐knife experimental vials, was irradiated on the same day using the Nucletron afterloader employing an I192r microSelectron source to deliver 10 Gy at a distance of 1 cm along the transverse bisector of the source in its single dwell position (thus delivering a dose ranging from 2.5 Gy at 2 cm to about 100 Gy at 3 mm and providing ample data for calibration purposes in a single gel vial/irradiation).

### C. Gel dosimetry

Two days postirradiation, the gel vials were imaged on the same MR scanner used to provide the images imported to the TPS for planning purposes. A volume‐selective 32‐echo Carr‐Purcell‐Meiboom‐Gill pulse sequence was used (TE1, TE2,…, TE32=40 ms, 80 ms,…, 1280 ms, TR of 2.3 s, reconstructed voxel size of $1\times 1\times 1\;{\text{mm}}^{3}$), with phase encoding being applied in two orthogonal directions and Fourier interpolation taking place in the slice reconstruction direction.^(15)^ After discarding the first echo of the 32‐echo train, a single T2 map (an image on which pixel signal intensity represents the nuclear magnetic resonance spin‐spin relaxation time T2 of the corresponding gel voxel) was automatically derived for each reconstructed slice. These maps were exported from the scanner in DICOM‐3 format and then imported into MatLab v6.5 (The Mathworks, Natick, MA) to construct a 3D T2 matrix, which was subsequently converted to an $R2_{\text{NET}}(=1/T2)$ relaxation rate matrix by subtraction of the zero dose reading (R2(0)) acquired by the corresponding control gel scanned in the same imaging session.

**Figure 1:**
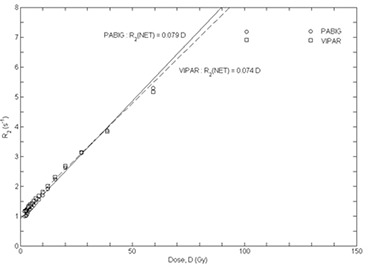
PABIG and VIPAR gel calibration curves

A linear calibration curve from 0 Gy to 40 Gy, presented in Fig. 1, was derived for each of the manufactured gel batches using the $R2_{\text{NET}}$ values of the corresponding brachytherapy irradiated samples^(13)^:

$$\begin{array}{l} R2_{\text{NET}}(D)=\text{aD}=(0.079\pm 0.006)\;D,r^{2}=0.994\;\text{for}\;\text{PABIG}\;\text{gels}\\ {\text{R2}}_{\text{NET}}(D)=\text{aD}=(0.074\pm 0.004)\;D,r^{2}=0.994\;\text{for}\;\text{VIPAR}\;\text{gels} \end{array}$$

**Figure 2:**
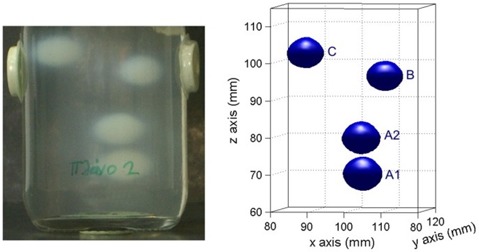
Presentations of gel irradiations and 3D dose distributions. Left: A photograph of the VIPAR gel–filled vial irradiated with a Leksell gamma knife C unit resembling treatment of four metastases with four 8‐mm collimator shots. Right: A 3D plot of the 50% isodose for each target as measured with the VIPAR polymer gel–MRI method.

**Figure 3:**
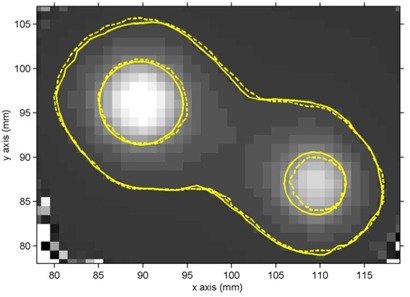
Dose distribution comparison in an axial plane. Comparison of PABIG polymer gel–MRI measured (solid outline) and GammaPlan calculated (dashed outline) relative dose distributions of 20% and 50% on an axial plane at z=101 mm containing targets B and C.

**Figure 4:**
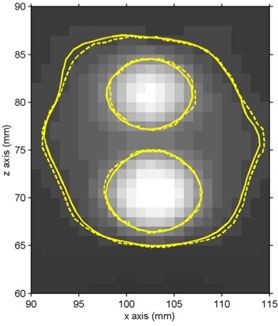
Dose distribution comparison in a coronal plane. Comparison of PABIG polymer gel–MRI measured (solid outline) and GammaPlan calculated (dashed outline) relative dose distributions of 20% and 50% on a coronal plane at y=99 mm containing targets A1 and A2.

Shot center localization in the experimental gel vials was performed on a subvoxel scale by exploiting the symmetry of the full 3D T2 distribution measured for each shot. In brief, 3D objects were created in the T2 matrix by setting measurement voxel T2 values equal to unity or zero, depending on whether they were lower or greater, respectively, than a set T2 threshold value. The center of mass and the axis of symmetry of the 3D objects were then calculated. This procedure was repeated for a number of different threshold values (> 5), which corresponded to doses near the 50% isodoses where the dose distributions are symmetrical (see also Figs. 2 to 4, where the 50% isodose is highlighted). Results correspond to independent estimates averaged to determine the coordinates of each shot center with an uncertainty equal to the corresponding standard deviation.

For the comparison of experimental data derived as described above with corresponding TPS calculations, the TPS output for selected isodose lines on specific axial, coronal, and sagittal planes had to be digitized, since the export of the raw TPS‐calculated data in a 3D grid suitable for comparison with the 3D matrix of experimental relative dose data is prohibited by the manufacturer's policy not to publicize the format of the binary data stored in the patient database of the gamma‐knife unit.

## III. RESULTS

Figure 2 (left) presents the VIPAR‐filled gel vial irradiated with four 8‐mm collimator shots according to the GammaPlan TPS‐generated multiple metastases treatment plan. In the same figure (right) the corresponding relative 3D relative dose distribution, measured using polymer gel dosimetry, is also presented in the form of isosurfaces of the prescription isodose for each of the four targets. This presentation was chosen to depict the relative extent of each target and to allow for a gross inspection of the geometrical accuracy in the delivery of the four 8‐mm shots by comparison of the observed location of each shot to its center coordinates in the treatment plan.

Quantitative results for the mechanical accuracy derived as described above are presented in Table 1 for both gel formulations used herein. In this table it can be seen that differences between experimentally derived and TPS‐reported shot center coordinates is well within the experimental uncertainty of one imaging pixel in the MRI gel readout session (< 1 mm).

**Table 1:** Shot center determinations. A comparison of the nominal Cartesian coordinates of the center of the four targets in the multiple metastasis treatment plan generated using the GammaPlan TPS software with corresponding experimental results measured using the VIPAR and PABIG gel formulations irradiated on a Leksell Gamma Knife model C unit. The maximum dose measured at each target is also shown in the last column (nominal: 20 Gy for PABIG and 30 Gy for VIPAR).

		*x*‐axis (mm)	*y*‐axis (mm)	*z*‐axis (mm)	Dose (Gy)
A1	TPS	103.3	100.7	70.3	
	PABIG	103.59±0.05	100.62±0.02	70.55±0.02	18.18±0.66
	VIPAR	104.00±0.02	101.56±0.04	71.04±0.05	28.09±1.03
A2	TPS	103.3	94.5	80.9	
	PABIG	103.26±0.03	94.38±0.03	80.82±0.04	18.55±0.67
	VIPAR	103.52±0.02	94.80±0.04	80.87±0.03	28.40±1.04
B	TPS	110.2	87.2	97.9	
	PABIG	109.81±0.02	86.97±0.02	97.88±0.02	18.30±0.66
	VIPAR	109.71±0.05	86.77±0.02	97.64±0.02	27.62±1.01
C	TPS	90	95.7	104.1	
	PABIG	90.05±0.05	96.13±0.04	103.94±0.03	18.39±0.67
	VIPAR	89.47±0.03	94.98±0.04	103.65±0.03	26.49±0.98

The maximum dose delivered to the center of each shot, which also corresponds to the maximum dose measured at each target, was determined by averaging the values of a number of voxels (> 20) in the dose plateau region of each shot,^(10)^ and it is also presented in Table 1. Comparison with corresponding TPS calculations (20 Gy for PABIG and 30 Gy for VIPAR) shows that measured dose values are lower by 9%, on average, for both gel formulations. This dose underestimation is mainly due to the Plexiglas used in the irradiation setup. Indeed, Monte Carlo simulations have shown that although Plexiglass is an acceptable material for relative dosimetry,^(16)^ it leads to a dose underestimation of the order of 8% in absolute dose measurements within the used Plexiglas phantom for the 8‐mm collimator helmet relative to measurements in water or water‐equivalent phantom materials.^(17)^ Moreover, there is a corresponding underestimation in the dose due to the presence of Pyrex glass of the gel vial (0.25 cm thick), which is of the order of 1.5%.

Given the proximity of the four collimator shots (see Table 1 and Fig. 2), it is expected that the isodose lines around a specific target will be distorted from the spherical shape expected from a single shot. This is especially true for the relatively lower isodose values, such as the 20% isodose line, which are potentially closer to a neighboring shot center. For the used irradiation pattern, these distortions are greatest between targets A1 and A2 (where Δx=0 mm, Δy=6.2 mm, and Δz=10.6 mm) and between targets B and C (where Δx=20.2 mm, Δy=8.5 mm and Δz=6.2 mm). Figures 3 and 4 present indicative PABIG‐gel measured and GammaPlan‐calculated percentage isodose contours of 20% and 50% superimposed on the corresponding T2 MR images. Figure 3 corresponds to an axial plane including both B and C targets, while Fig. 4 corresponds to a coronal plane including both A1 and A2 targets. In both figures close agreement is observed between measured and calculated results with average distance to agreement well under the experimental uncertainty of one imaging pixel in the MRI gel readout session (1 mm). Distance to agreement was preferred over absolute dose differences in the above comparison in view of the steep dose gradients met in gamma‐knife applications that result in dose uncertainty on the order of 10% near the 50% isodose due to the imaging pixel size dimensions of 1 mm.^(18)^ Corresponding measurements using the VIPAR gel formulation exhibited a corresponding average agreement with TPS calculations, although compared with PABIG results, they present a significant statistical fluctuation, especially for the lower percentage measured isodose line of 20%. This statistical uncertainty is the combined effect of increased statistical uncertainty in the low‐dose region, mainly due to the noise in T2 measurement,^(19)^ and dose resolution of the particular gel formulation.^(^ ^20^ ^,^ ^21^ ^)^ The latter is directly related to the noise in the T2 image as well as the dose sensitivity of the gel used and becomes a limiting factor as one moves toward the low‐dose gradient region.

## IV. CONCLUSIONS

The polymer gel–MRI dosimetry method was used in this work for the 3D verification of gamma‐knife treatment of multiple targets using two different gel formulations. In these applications, factors such as different prescription isodoses, maximum dose, and/or increased intratarget distances necessitate treatment‐planning system calculations in different dose matrixes to be combined in the final stage of the calculations. Experimental results were compared to corresponding treatment‐planning calculations in the form of relative dose distributions, and agreement within experimental uncertainties was observed. In addition, mechanical accuracy of such applications was verified using a procedure that determines the coordinates of each shot center from the symmetry of the full 3D T2 distributions measured for each shot. Overall, polymer gel dosimetry was found capable of verifying the complete chain of radiosurgery treatment in gamma‐knife applications involving the irradiation of multiple targets.

## ACKNOWLEDGMENTS

This work was supported by a PYTHAGORAS II research grant by the Greek Ministry of National Education and Religious Affairs within the framework of the EPEAEK II program.
